# Success rate of fractured teeth receiving modified crown lengthening surgery and restorations

**DOI:** 10.1186/s12903-022-02143-z

**Published:** 2022-03-30

**Authors:** Cui Wang, Xue-ting Jia, Min Zhen, Wen-Jie Hu, Hao Zhang, Kwok-Hung Chung

**Affiliations:** 1 Department of Periodontology, Peking University School and Hospital of Stomatology & National Clinical Research Center for Oral Diseases & National Engineering Laboratory for Digital and Material Technology of Stomatology & Beijing Key Laboratory of Digital Stomatology, 22 Zhongguancun S Ave, Haidian District, Beijing, 100081 China; 2 Department of Restorative Dentistry, Peking University School and Hospital of Stomatology & National Clinical Research Center for Oral Diseases & National Engineering Laboratory for Digital and Material Technology of Stomatology & Beijing Key Laboratory of Digital Stomatology, Beijing, China; 3 Department of Restorative Dentistry, School of Dentistry, University of Washington, Seattle, WA USA

**Keywords:** Structurally compromised teeth, Odontoplasty, Root reshaping, Success time, Success rate

## Abstract

**Background:**

Whether to preserve a structurally compromised tooth or remove it is a dilemma often encountered by clinicians. The aim of this study was to assess the long-term success rate of fractured teeth preserved by modified crown lengthening surgery and restorations.

**Methods:**

Thirty-nine patients with a total of 45 fractured teeth who had received modified crown lengthening surgery were recruited and examined. Numbers of teeth lost were recorded, and the criteria for successful teeth were defined. Kaplan–Meier estimator was used to determine the success rate. Possible risk factors were compared between successful and unsuccessful groups by a Cox regression analysis to explore the potential predictors of failure with a significant level at α = 0.05.

**Results:**

The mean ± SD of success time without considering variants was 6.2 ± 0.6 years (95% CI 5.1–7.7). The mean survival rates ± SD at 1.0-, 2.0-, 3.0-, 5.0-, 7.0-, and 9.0-year intervals was 97.8 ± 2.2%, 92.2 ± 4.4%, 72.8 ± 7.9%, 68.2 ± 8.6%, 60.7 ± 10.5%, and 40.4 ± 13.6%, respectively. Failure cases in teeth with poor plaque control and step-shaped fracture margin were significantly more than those with good plaque control and knife-shaped fracture margin (HR = 7.237, *p* = 0.011; HR = 15.399, *p* = 0.006; respectively).

**Conclusions:**

Fractured teeth treated with modified crown lengthening surgery are anticipated to have a high clinical success rate for 6.2 ± 0.6 years. Plaque control and fracture morphology appeared to be significantly associated with the success of the multidisciplinary treatment approach.

## Background

Maintenance of the natural dentition with adequate function and desirable esthetics has long been a key therapeutic goal of the current evidence-based dentistry [1–3]. When traumatic injury occurs to natural dentition, dentists and patients face a challenging dilemma to treatment plan a structurally compromised tooth. Priority is always given to preserve a structurally compromised tooth after comprehensively multi-factorial risk assessments with respect to its irreplaceable nervous perception and psycho-social effects [4, 5]. In most of the cases, multidisciplinary treatment including root canal therapy, pre-restorative crown lengthening surgery before definitive restoration procedures are indicated [6–10].

**Fig. 1:**
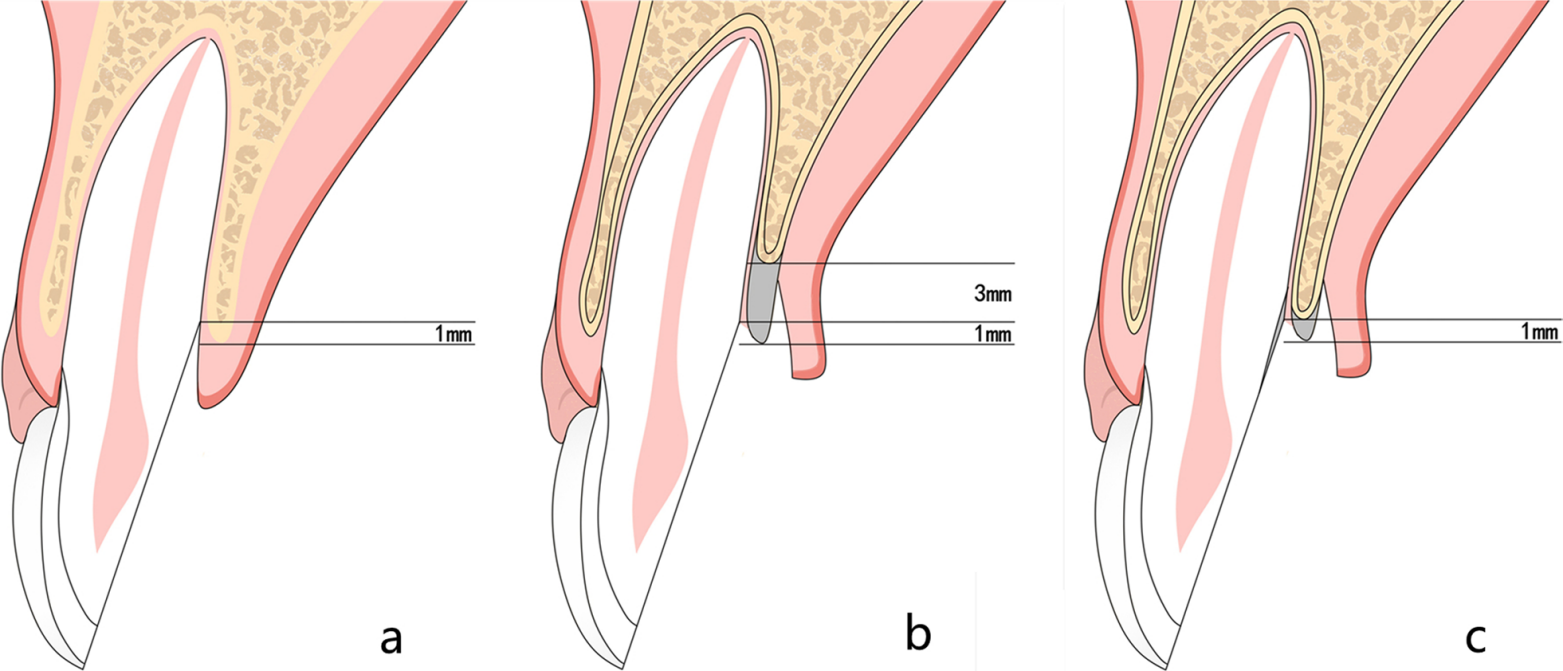
The schematic diagram for modified crown lengthening procedures in different scenarios. **a** Tooth is diagnosed with crown-root fracture with its margin 1 mm underneath the alveolar crest. **b** Removal of 4 mm bone tissue to create new biologic width in conventional procedures. **c** Approximately 1 mm bone reduction to expose fracture margin to form smoothy root surface and eliminate the fracture margin in modified procedures

The purpose of a surgical crown lengthening procedure is to provide adequate supra-crestal tooth structure for the future restoration to maintain periodontal health and avoid biological width violation. However, conventional crown lengthening surgery (CCLS) usually involves a bone resection procedure in order to create a minimum of 3 mm between the restorative finish line and the alveolar bone crest [7, 11, 12]. For cases with extensive sub-gingival fractures, aggressive ostectomy is sometimes needed to expose the fracture margin during CCLS. Consequently, inadequate bone support may result, and the structurally compromised tooth would be at risk for failure [6, 7, 12–14]. Modified crown lengthening surgery (MCLS) has been recommended and practiced clinically for more than 20 years; it involves the odontoplasty technique, enabling the re-establishment of the biologic width with less bone resection needed to expose enough tooth structure, Fig. 1 [15–18]. Odontoplastic procedures aim at reshaping the neighboring area of fracture margin to form a smooth root surface and shift the fracture margin coronally. The benefit of the modified odontoplasty technique is to minimize alveolar bone resection, which positively improves prognosis of the structurally compromised teeth. In addition, more alveolar bone preservation will help to maintain the integrity of the extraction site for the future oral rehabilitation procedures [15–18]. Short-term clinical outcomes of CCLS including periodontal indices, changes of free gingival margin, crown length, and bone level, have been reported [19–23]. The long-term outcomes of teeth preserved after CCLS are sparse [8, 20, 24].

A few studies with small sample sizes reported that MCLS achieved a good clinical outcome, which can be a feasible and minimally invasive therapeutic alternative for management of structurally compromised teeth [16, 17, 22]. To our knowledge, there is no long-term data available on the success rate of fractured teeth receiving MCLS and definitive restorations. Therefore, the aim of this study was to retrospectively evaluate the success rate of structurally compromised teeth after receiving multidisciplinary treatment including MCLS, root canal therapy, and crown restorations. Along with this, the predictive factors for failure are also investigated to assist the clinicians for evidence-based decision making.

## Methods

### Subject selection and sampling

This retrospective study was approved by the Medical Ethical Committee for Human Investigations of Peking University Health Science Centre, Beijing, China (No. PKUSSIRB-2012067) and adhered to the World Medical Association Declaration of Helsinki guidelines. Medical records of all patients who suffered from clinical crown fracture and received MCLS combined with crown restorations by the same clinician (WH) from July 2004 to June 2013 at the research institute were reviewed. All fractured teeth were prepared with feather-edge finish line equagingivally or 0.5–1.0 mm subgingivally at the fracture line region and chamfer finish line design for the remained tooth structure. Crown restoration was started approximately 3 to 6 months after MCLS procedure. The definitive crown was fabricated with a porcelain-fused-to-metal crown using noble metal alloy (The Argen Corporation, CA, USA) and cemented with luting cement (RelyX Luting, 3 M ESPE, St. Paul, MN, USA). Clinical data and information of all the subjects for possible inclusion were scanned and analyzed. Patients were excluded from the study if they meet one or more of the following criteria: (1) Less than 1-year follow-up time; (2) Incomplete clinical information for the last follow-up visits; (3) Patients who had systemic disease affecting periodontal health, including pregnant women. Moreover, teeth were eligible if they meet all the following criteria: (1) Periodontally healthy before MCLS with probing depth (PD) ≤ 3 mm and no bleeding on probing, no mobility and furcation involvement, continuous and clear lamina dura with no widen periodontal space revealed from radiographic examination; (2) Successful root canal treatment or retreatment before MCLS; (3) Good marginal adaptation with proper interproximal and occlusal contacts; and (4) crown to root ratio was ≤ 1 immediately after definitive restoration.

### Data collection

The initially selected patients were called in and informed of details of this study. Written consent was signed, and clinical and radiographic examinations were performed for those who agreed to participate in the investigation. All examinations were conducted by the same senior clinician (WH), who also performed the MCLS procedures. The following data were recorded: plaque index (PLI), bleeding on probing (BOP), probing depth (PD), position of the restoration margin relative to gingival margin (RM-GM) at six sites using a Williams periodontal probe, furcation involvement (FI) [25], and tooth mobility [26] of both the examining teeth and the contralateral teeth. Patient’s subjective perception of the affected tooth was recorded, including swollen gums, gum hemorrhage or exudate, tooth mobility and/or displacement, and toothache and/or chewing discomfort. Patients’ satisfaction for function and esthetics about the treated teeth were assessed using visual analog scale (VAS). All the perception data were collected by questionnaires. A periapical radiograph of each treated tooth was taken and compared with the pre-surgical radiograph (baseline) to examine the density of lamina dura, bone height, periodontal space of the treated teeth changed, and periapical lesion or root fracture occurred. In addition, demographic data including age, gender, smoking status, systemic health, follow-up time, adverse oral parafunction (i.e. bruxism, clenching, unilateral mastication, and so on), and periodontal maintenance were collected. Details of the selected teeth before and during MCLS including tooth location, morphology of initial fracture margin (MFM), location of fracture margin relative to gingival margin (FM-GM), quantity of bone resection, and any residual fragment existed were assessed. Grading criteria were specified and applied for each of the above variables (Table 1).

**Table 1:** Variables and the grading criteria involved in data analysis

Variables	Grading scale
Age	Years old
Gender	Female = 0; Male = 1
Tooth position	Anterior = 0; Posterior = 1
Location of fracture margin	Supra/equal-crestal = 0; Sub-crestal = 1
Morphology of fracture margin	Knifed = 0; Stepped = 1
Residual fragments	Yes = 0; No = 1
Quantity of bone resection	Millimeters (mm)
RM-GM	Millimeters (mm)
Plaque control	Good = 0; Poor = 1
Smoking status	No = 0; Yes = 1
Adverse oral parafunction	No = 0; Yes = 1
Failure	Yes = 0; No = 1
Follow-up time	Years

Residual fragments: split pieces of tooth substance found during MCLS; RM-GM: position of the restoration margin relative to gingival margin

A restored fractured tooth was defined as having success if it met all of the following criteria: (1) Survival of the teeth with the definitive restorations without subjective symptoms along with both esthetic and functional VAS score ≥ 8 [16]; (2) PD ≤ 5 mm, tooth mobility ≤ I degree, no furcation involvement or ≤ II degree furcation involvement that can be controlled by initial periodontal therapy; (3) Clearness and continuity of lamina dura radiographically without progressive bone loss and widening periodontal space; (4) No secondary caries, extensive periapical lesion, sinus tract, and root fracture were detected. Failure was deemed if a tooth was lost during the follow-up time or met anyone of the previously mentioned items.

### Statistical analysis

The data were analyzed using the SPSS 24.0 (SPSS Inc, Chicago, IL, USA). Descriptive statistical analysis of demographic data was performed using mean ± standard deviation or constituent ratios for measurement or enumeration data, respectively. A Kaplan–Meier analysis was used to calculate the success rate. To compare the equality of success distributions relative to gender, tooth type, status of plaque control (PLI ≤ 1 was defined as good, PLI > 1 was defined as poor plaque control), morphology of fracture margin, residual fragments, smoking status by Tarone-Ware test. Cox-regression was performed to determine predictors for success. A statistically significant level was set at *p* < 0.05.

## Results

**Fig. 2:**
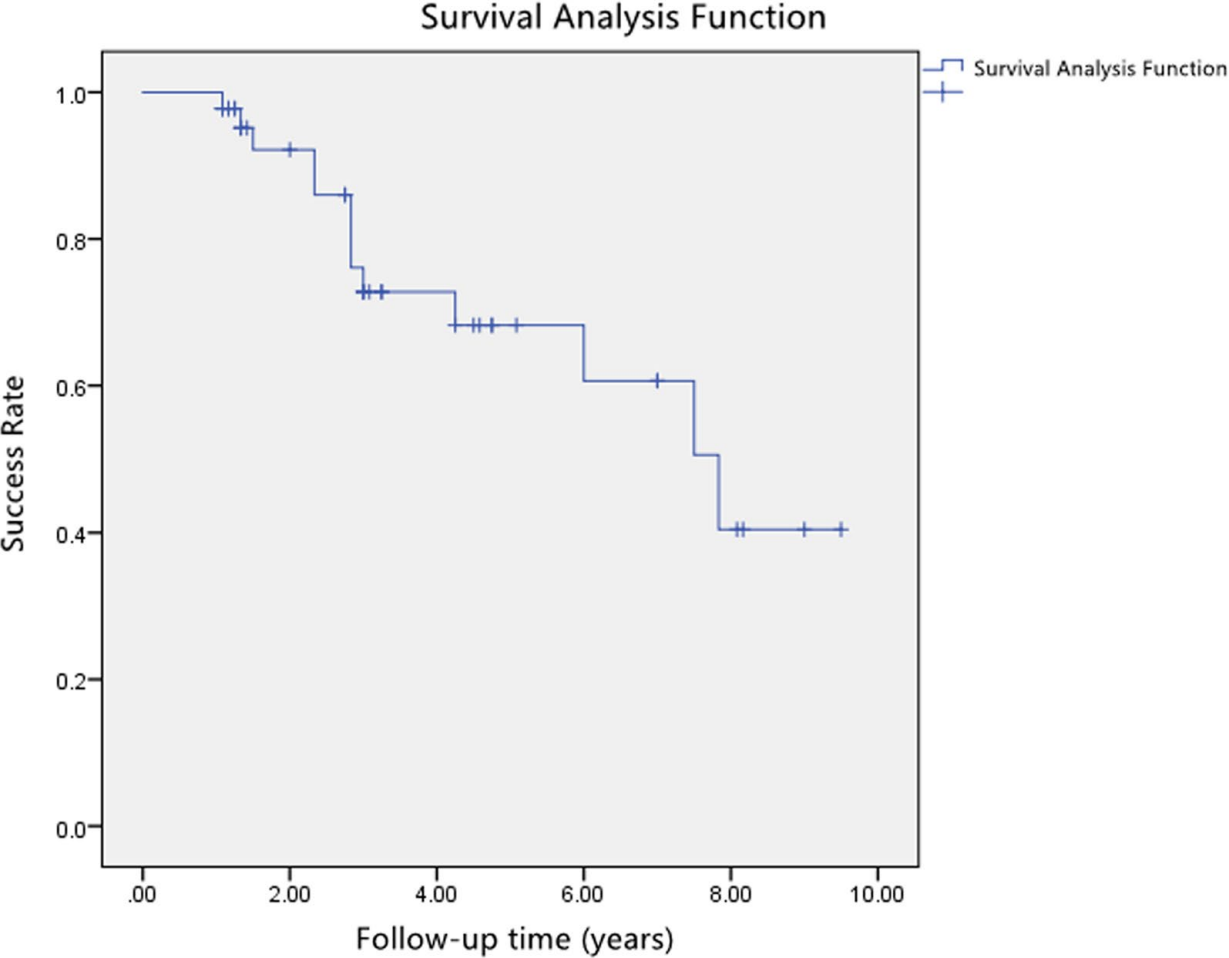
Cumulative success rates of modified crown lengthening surgery and restorations relative to time

**Fig. 3:**
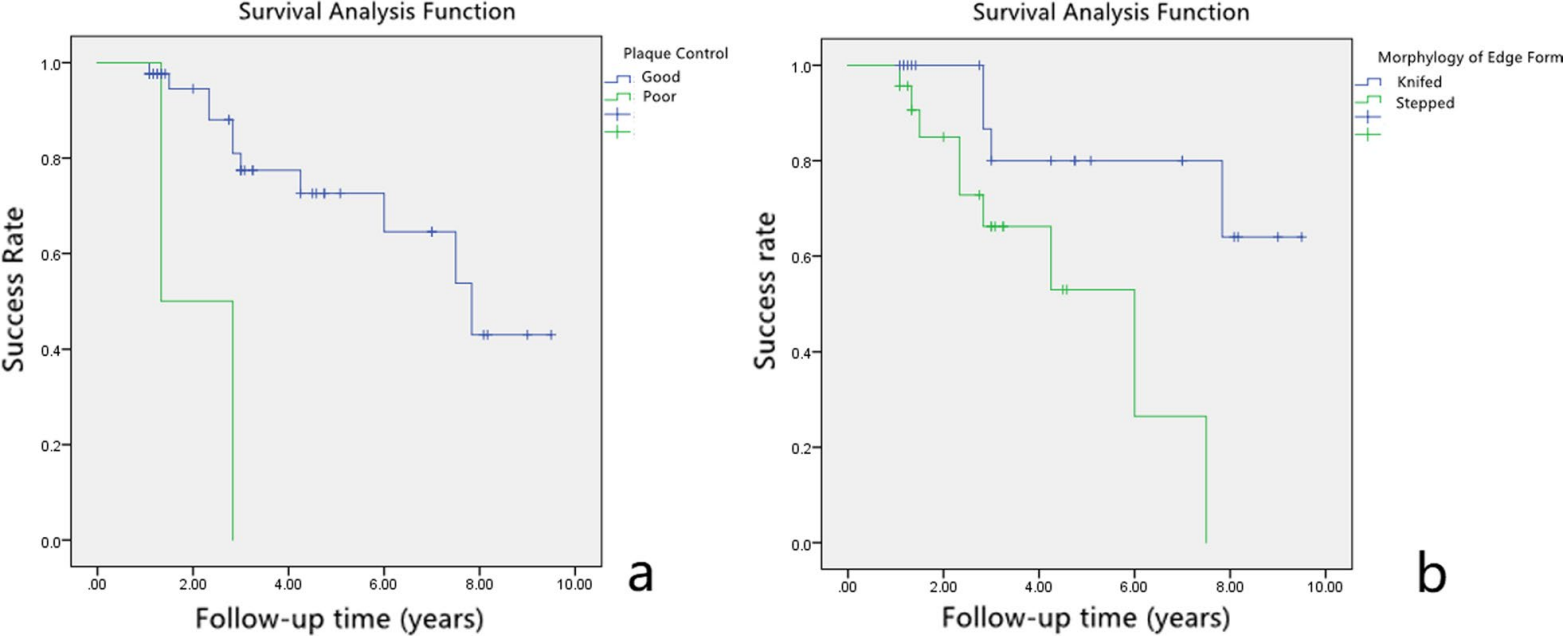
Kaplan–Meier curves of cumulative success rates for **a** good or poor plaque control, and **b** knifed- or stepped- fracture margin

A total of 57 patients with 67 teeth receiving MCLS were screened. Twenty-two teeth were excluded because they had follow-up times less than 1 year. A total 45 teeth were recruited from 39 patients, including 23 (59.0%) males and 16 (41.0%) females, with a mean age of 37.6 ± 13.1 years old, ranging from 16–76 years (Table 2). Only a left lower first molar tooth restored with cast post-crown was extracted at the 3-year follow-up due to vertical root fracture. Thirty-two cases were determined as successful. The Kaplan–Meier analysis showed success rates at 1.0-, 2.0-, 3.0-, 5.0-, 7.0- and 9.0-year follow-up, listed in Fig. 2 and Table 3. Complications for failed cases at the time of follow-up examinations were listed in Table 4. The years of success relative to gender, tooth location, status of plaque control, morphology of fracture margin, residual fragments, and smoking status are shown in Table 5. Patients who had meticulous plaque control achieved longer years of success than those whose plaque control were poor (7.0 ± 0.6 years versus 2.1 ± 0.8 years, *p* = 0.002). Moreover, teeth with knife-shaped morphology of fracture margin survived more years without complications than those with step-shaped margin (7.9 ± 0.7 versus 4.8 ± 0.7, *p* = 0.028) (Fig. 3). There were no statistically significant differences in mean of years of success in terms of gender, tooth type, residual fragments, and smoking status (*p* > 0.05).

**Table 2:** Demographic data of 39 subjects and 45 sample teeth

Subjects	
Age	37.6 ± 13.1(16.0–76.0) years
Gender (Male/Female)	23 (59.0%)/16 (41.0%)
Smoking status (Yes/No)	7 (17.9%)/32 (82.1%)

Teeth sample	
Tooth type (anterior/posterior)	27 (60.0%)/18 (40.0%)
Follow-up time	3.6 ± 2.5 (1.1–9.5) years

**Table 3:** Success rate for sample teeth. (Means ± Standard Deviation, %)

Time (years)	1.0-	2.0-	3.0-	5.0-	7.0-	9.0-
Success rate	97.8 ± 2.2	92.2 ± 4.4	72.8 ± 7.9	68.2 ± 8.6	60.7 ± 10.5	40.4 ± 13.6

**Table 4:** Complications of failure teeth at the time of examination

Complications	Number	Percentage of all sample
Periodontal failure	8	17.8
Endodontic therapy	1	2.2
Prosthodontic complications	0	0
Subjective symptoms	1	2.2

**Table 5:** Mean ± standard deviation of years of success relative to different variables

Variables	Status	Years of success	χ^2^ value	*p* value
Gender	Male	6.5 ± 0.6	0.040	0.841
	Female	6.4 ± 1.0		
Tooth type	Anterior	6.3 ± 0.6	0.080	0.777
	Posterior	6.5 ± 0.8		
Plaque control	Poor	2.1 ± 0.8	9.559	0.002*
	Good	7.0 ± 0.6		
Morphology of fracture margin	Stepped	4.8 ± 0.7	4.799	0.028*
	Knifed	7.9 ± 0.7		
Residual fragments	Yes	5.5 ± 0.8	1.066	0.302
	No	7.4 ± 0.8		
Smoking status	Yes	5.0 ± 1.0	2.875	0.090
	No	7.5 ± 0.7		
Adverse oral parafunction	Yes	3.2 ± 0.3	2.868	0.090
	No	7.1 ± 0.6		

*Significant at *p* value < 0.05

Cox univariate regression analysis showed *p* values with respect to gender, age, FM-GM, quantity of bone resection, RM-GM, plaque control, tooth type, morphology of fracture margin, residual fragments, smoking status, and adverse oral parafunction were presented in Table 6. Table 7 indicates the hazard ratio of various studied variables. Failure cases in patients with poor plaque control were approximately 15 times more than in patients with good plaque control (*p* < 0.05). Teeth with step-shaped fracture margin had approximately 7 times more failure rate than those with knife-shaped ones (*p* < 0.01).

**Table 6:** Results of Cox univariate regression analysis

Variables	Hazard Ratio	SE	95% CI	*p* value
Gender	1.024	0.579	0.329–3.188	0.968
Age	0.970	0.026	0.922–1.020	0.235
Location of fracture margin	1.792	0.780	0.388–8.267	0.455
Quantity of bone resection	0.591	0.419	0.260–1.343	0.209
RM-GM	0.923	0.393	0.427–1.995	0.838
Plaque control	8.277	0.819	1.663–41.204	0.010*
Tooth type	1.198	0.560	0.400–3.588	0.747
Morphology of fracture margin	4.825	0.687	1.255–18.550	0.022*
Residual fragments	2.256	0.661	0.617–8.247	0.219
Smoking status	3.151	0.560	1.052–9.439	0.040*
Adverse oral parafunction	3.368	0.701	0.852–13.317	0.083

*Significant at *p* value < 0.05

**Table 7:** Results of Cox regression estimates

Variables	Hazard Ratio	SE	95% CI	*p* value
*Morphology of fracture margin*				
Stepped–Knifed ratio	7.237	0.774	1.586–3.019	0.011*
*Plaque control*				
Poor–Good Ratio	15.399	0.991	2.206–107.499	0.006*
*Smoking status*				
Yes–No Ratio	2.000	0.598	0.619–6.462	0.247

*Significant at *p* value < 0.05

## Discussion

This retrospective study aimed to evaluate the success rate of fractured teeth treated with MCLS combined with root canal therapy and restorations. In the present investigation, we defined a case of success as having no complications or minimal complications that could be controlled by non-surgical procedures. One failure case (a lower left first molar) was deemed hopeless because of vertical root fracture after 3 years of function. It has been widely documented that root canal instrumentations and restoration of post-endodontically treated teeth usually decrease resistance to tooth fracture [27, 28]. In addition, intra-canal post insertion was demonstrated to be associated with increased rate of vertical root fracture [29, 30]. In addition, traumatic teeth usually consist of crazing or cracks which were undetectable initially. The residual cracks can be provoked and will start propagating during surgical intervention and prosthodontic management, especially during functional loading. Close observation and managed conservatively should be the best policy in clinical practice especially for the cases after MCLS procedure.

To our knowledge, only case reports or investigations with small sample size reported clinical outcomes of MCLS [10, 16, 17, 31–33]. In this study, the success rate of teeth preserved by MCLS and crown restoration was 97.8% for the first 1-year recall and decreased into 40.4% at the 9-year follow-up, mainly related to periodontal complications. Da Cruz et al. [17] reported that 12 out of 14 cases presented total success, while two other cases presented relative success of odontoplasty during CCLS with a mean of 13.57 months follow-up period respectively; this was close to the 1.0-year success rate reported in our study.

MCLS involves the combination of CCLS with minimum odontoplasty technique which brings the benefit of less bone resection of the structurally compromised teeth. However, minimizing the remaining tooth structure during odontoplasty procedure especially in the worn area of the structurally compromised tooth may subject to a higher risk of secondary fracture. Results of a previous study using finite element analysis to investigate crown-root fracture cases restored with post-core restoration demonstrated that stress concentration exists in the worn area [34]. Therefore, establishing a ferrule to decrease occlusal stress and maintain retention of the restoration at the worn area is important when the definitive restoration combined with casting post-core is designed and inserted. The actual effect of odontoplasty on the success rate of teeth receiving MCLS remains unclear. Moghaddam et al. [8] reported survival rate ranging from 83.1 to 98.3% from 1 to 10 years for teeth treated with CCLS combined with root canal therapy and prosthodontic treatments[8]. Sajjad Ashnagar et al. [24] reported that structurally compromised teeth have a reasonable long-term survival rate close to 80% after 10 years and patients with high fracture or caries risk may pose a higher chance of failure. Unfortunately, results of this current study cannot compare with previous studies because of differences of the criteria of case selection and success. In the future, randomized controlled clinical studies with larger sample sizes and a longer follow-up period will be designed to clarify the impact of MCLS on the success rate of structurally compromised teeth.

**Fig. 4:**
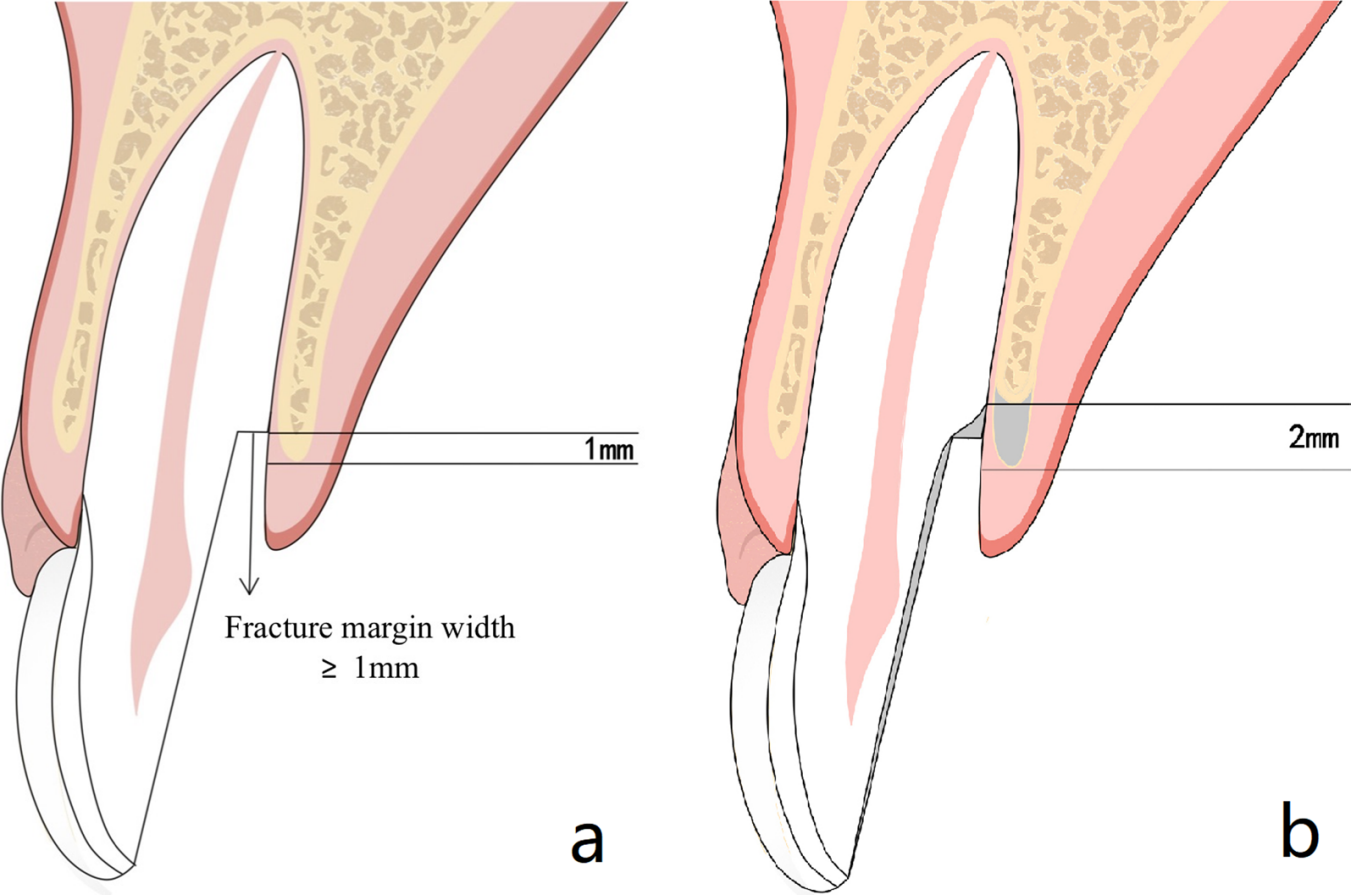
**a** Stepped fracture root margin. **b** More reduction of alveolar bone and root structure to achieve a smooth root surface

The morphology of fracture margin and plaque control exercise were proved to be the key factors of the success rate of the teeth treated with MCLS in this study. Therefore, patient selection and motivation also play an important role in obtaining the desired and anticipated outcome in clinical practice. Teeth with knife-edged fracture margin and good plaque control during the follow-ups are more likely to be maintained successfully. It is speculated that knife-edged fracture margins are easier to obtain a line feed through odontoplasty with less dental tissue and alveolar bone sacrificed. While more alveolar bone height will be reduced to expose adequate root surface to perform odontoplasty and greater amount of tooth structure will be sacrificed to create a smoothy root surface or coronally shift the fracture margin (Fig. 4).

Another alternative treatment approaches for compromised teeth with extensive fracture would be extraction of the teeth and replaced with an implant-supported prosthesis. Dental implant therapy may seem more predictable in replacing a compromised tooth. A systematic review by Holm-Pederson et al.[35] determined that dental implants achieved high success rates similar with periodontally compromised teeth which are treated and maintained regularly, but implants did not surpass the longevity of even compromised yet treated natural teeth. Therefore, it is of paramount importance to inform the patient of the prosthetic and biologic complications of implants in the long-term follow-ups. However, no absolute suggestions can be made, since there does not appear to be a successful difference between a crowned endodontically treated tooth and a single implant. Esthetics, function, comfort, cost-effectiveness, and patient’s desires should be also considered. Although MCLS procedures increase the overall treatment cost and time, the multidisciplinary treatment approach is generally less costly than an implant and acquires comparable treatment duration. There is no denying that teeth preserved through multidisciplinary treatment and multiple procedures may be at risk for root fracture, secondary caries or periodontal disease. It appears that the benefits outweigh its harms as result of preservation of remaining root structure, alveolar bone companied by papilla, periodontal pressure perception, and less adverse impact on the surrounding teeth. Previous investigations suggested that tooth extraction may insert adverse influence on patients’ psychological outcomes and oral health-related quality of life especially for young patients [36]. Therefore, priority in treatment planning should be given to conservation management of the affected tooth by multidisciplinary treatment procedures even if there is no consensus on the best treatment plan for fractured teeth.

It is worth mentioning that the same senior clinician performed all the MCLS procedures as well as examinations and parallel periapical radiographs were taken during the follow-up visits in this study, which may result in some risk of bias. Future studies will benefit from a research design with different examiners performing the exams. In addition, the results of the present study should be further evaluated in future investigations adopting standardized parallel periapical radiographs to make an accurate comparison with a larger sample size.

## Conclusions

Within the limitations of the current study, the following conclusions can be drawn:

1. MCLS procedures are clinically relevant in maintaining natural dentition.
2. The success rates for treated teeth are as high as 97.8% during the first 1-year recall period after crown restoration, but the rates decreased over time mainly related to periodontal complications.
3. Teeth treated with MCLS combined with crown restoration should be scheduled with close observation every 6-month for possible root fracture assessment and periodic supportive periodontal therapy to avoid severe periodontal complications.
4. Teeth with knife-edged fracture margins, usually caused by tooth injury, are good candidates for surgical procedures. Good plaque control should be addressed and emphasized during follow-up examination.

## Data Availability

The datasets used and analyzed during the current study are available from the corresponding author on reasonable request.

## Abbreviations

CCLS: Conventional crown lengthening surgery
MCLS: Modified crown lengthening surgery
PLI: Plaque index
BOP: Bleeding on probing
PD: Probing depth
RM-GM: Position of the restoration margin relative to gingival margin
FI: Furcation involvement
VAS: Visual analog scale
MFM: Morphology of initial fracture margin
FM-GM: Location of fracture margin relative to gingival margin
